# Overview of in vivo and ex vivo endpoints in murine food allergy models: Suitable for evaluation of the sensitizing capacity of novel proteins?

**DOI:** 10.1111/all.13943

**Published:** 2019-07-09

**Authors:** Laure Castan, Katrine L. Bøgh, Natalia Z. Maryniak, Michelle M. Epstein, Sahar Kazemi, Liam O'Mahony, Marie Bodinier, Joost J. Smit, Jolanda H. M. van Bilsen, Carine Blanchard, Robert Głogowski, Hana Kozáková, Martin Schwarzer, Mario Noti, Nicole de Wit, Grégory Bouchaud, Shanna Bastiaan‐Net

**Affiliations:** ^1^ INRA, UR 1268 BIA Nantes Nantes France; ^2^ National Food Institute Technical University of Denmark Kgs. Lyngby Denmark; ^3^ Experimental Allergy Laboratory, Department of Dermatology Medical University of Vienna Vienna Austria; ^4^ Department of Medicine, APC Microbiome Ireland National University of Ireland Cork Ireland; ^5^ Department of Microbiology, APC Microbiome Ireland National University of Ireland Cork Ireland; ^6^ Institute for Risk Assessment Sciences Utrecht University Utrecht The Netherlands; ^7^ TNO Zeist The Netherlands; ^8^ Consultant London UK; ^9^ Department of Animal Breeding and Production Warsaw University of Life Sciences Warsaw Poland; ^10^ Institute of Microbiology Czech Academy of Sciences Nový Hrádek Czech Republic; ^11^ Institute of Pathology University of Bern Bern Switzerland; ^12^ Wageningen Food and Biobased Research Wageningen The Netherlands

**Keywords:** animal models, biomarkers, food allergy, prevention

## Abstract

Significant efforts are necessary to introduce new dietary protein sources to feed a growing world population while maintaining food supply chain sustainability. Such a sustainable protein transition includes the use of highly modified proteins from side streams or the introduction of new protein sources that may lead to increased clinically relevant allergic sensitization. With food allergy being a major health problem of increasing concern, understanding the potential allergenicity of new or modified proteins is crucial to ensure public health protection. The best predictive risk assessment methods currently relied on are in vivo models, making the choice of endpoint parameters a key element in evaluating the sensitizing capacity of novel proteins. Here, we provide a comprehensive overview of the most frequently used in vivo and ex vivo endpoints in murine food allergy models, addressing their strengths and limitations for assessing sensitization risks. For optimal laboratory‐to‐laboratory reproducibility and reliable use of predictive tests for protein risk assessment, it is important that researchers maintain and apply the same relevant parameters and procedures. Thus, there is an urgent need for a consensus on key food allergy parameters to be applied in future food allergy research in synergy between both knowledge institutes and clinicians.

**Abbreviations**
Ag: allergen
ALA: alpha‐lactalbumin
BAT: basophil activation test
BLG: beta‐lactoglobulin
BN: Brown Norway
DC: dendritic cell
e.c.: epicutaneously
ELISA: enzyme‐linked immunosorbent assay
GM‐CSF: granulocyte‐macrophage colony‐stimulating factor
i.c.: intracutaneous
i.d.: intradermally
i.g.: intragastric
i.n.: intranasal
i.p.: intraperitoneally
Ig: immunoglobulin
ILC: innate lymphoid cell
i.v.: intravenous
KSCN: potassium thiocyanate
NA: not analysed
NKT: natural killer T cell
OVA: ovalbumin
PCR: polymerase chain reaction
RBL: rat basophil leukemia
s.c.: subcutaneously
SPT: skin prick test
Treg: regulatory T cell
TSLP: thymic stromal lymphopoietin
WPC: whey protein concentrate
WPH: whey protein hydrolysate

## 1 INTRODUCTION

**Figure 1:**
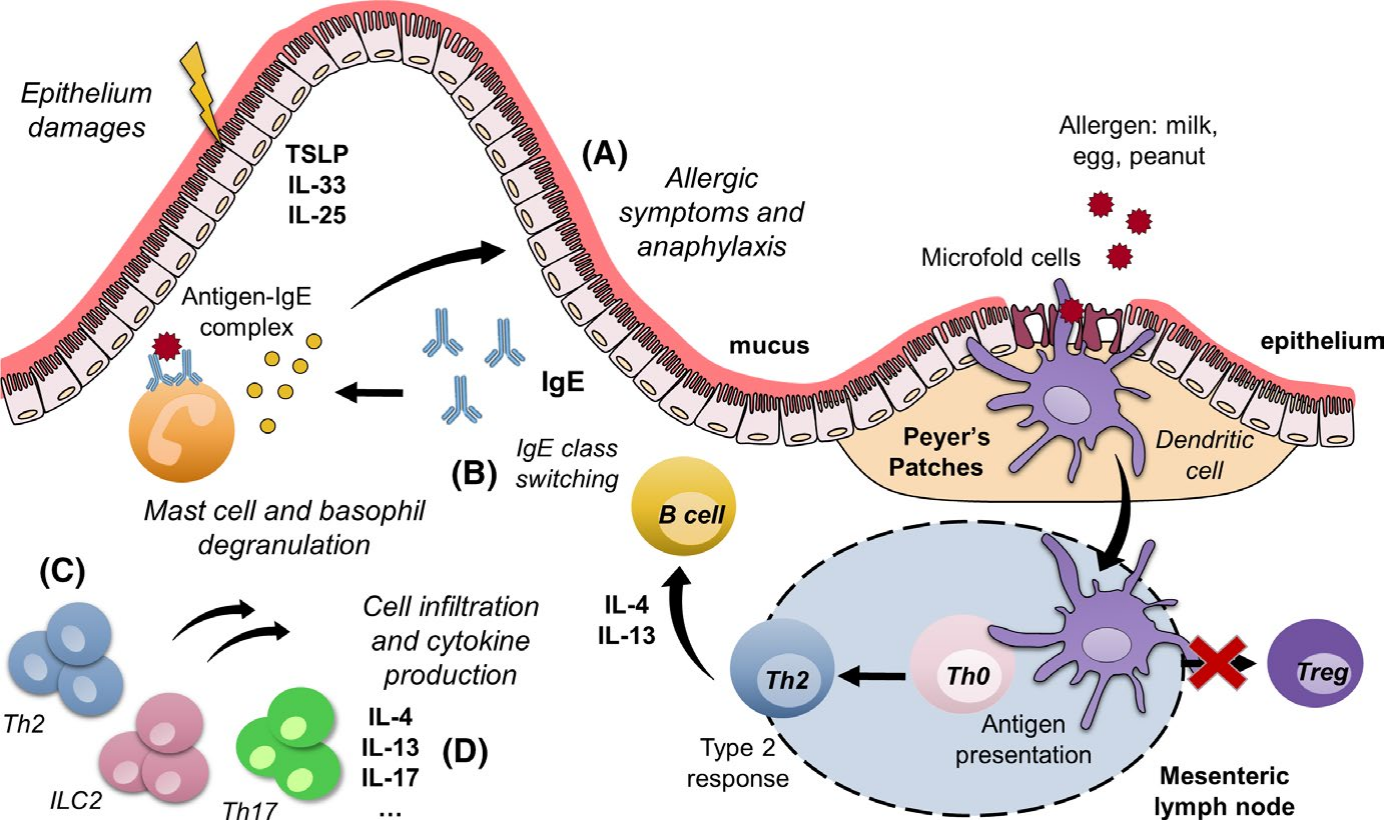
Immune mechanisms of food allergy and its associated principal measured endpoints. A, Assessment of allergic symptoms (body temperature) after allergen challenge. B, Evaluation of immunoglobulin (IgE) in serum. C, Phenotyping of T‐cell population. D, Cytokine production in response to allergen restimulation (ex vivo assay)

**Figure 2:**
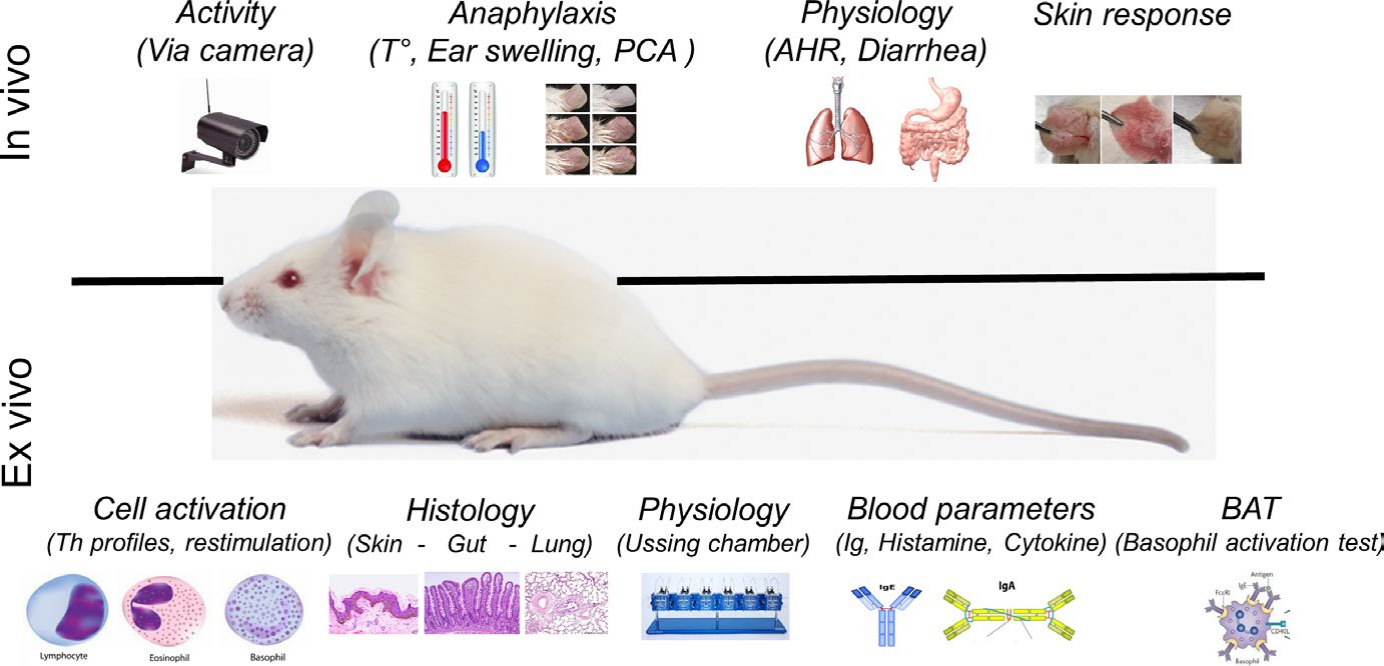
In vivo and ex vivo methodological endpoints used in murine food allergy models

A variety of in vitro and in vivo models have been developed that address the factors and mechanisms involved in the sensitization to food proteins.^1^, ^2^, ^3^, ^4^ Currently, approaches are being developed using protein chemistry and in vitro and in silico methods to characterize food proteins and derivatives that arise during product processing and reformulation, which may explain why certain food proteins induce sensitization of the immune system, while others are tolerated.^5^, ^6^ However, elucidating the mechanisms underlying allergen sensitization is a complex, multidimensional problem that often requires a wide range of additional in vivo and ex vivo experimentation,^5^ as a wide range of molecules, tissues, and cells play a role in the mechanisms underlying food allergen sensitization.^1^ For instance, epithelial release of thymic stromal lymphopoietin (TSLP), granulocyte‐macrophage colony‐stimulating factor (GM‐CSF), IL‐25, and IL‐33 upon local epithelial stress support type 2 helper T (Th2) cell pathology by attracting IL‐4 secreting lymphoid cells, basophils, and invariant natural killer T (iNKT) cells.^7^ Il‐4 promotes surface expression of Th2‐costimulatory molecule OX40 ligand on dendritic cells (DCs) 8 and cytokine secretion by Th2 lymphoid cells (ILC2s), which further augments DC activity and suppresses allergen‐specific regulatory T (Treg) cells.^9^, ^10^ This complexity, as depicted in Figure 1, illustrates the need for experimental food allergy models that integrate such complex cell‐tissue communication to assess the sensitization potential of new protein sources. Murine food allergy models, even though they have their limitations, are currently the best predictive models available to evaluate the food‐sensitizing capacity of new food proteins before introducing them into the human diet. Although researchers aim to reduce the use of experimental animals to address the 3R principle that guides animal experimentation to replace (alternative model), reduce (minimize number of animals), and refine (minimize animal pain and enhance animal welfare), there is a lack of replacement models such as in silico prediction models, in vitro primary cell assays, or tissue explants assays that are able to characterize and predict the human responses to food proteins. In the past, numerous experimental food allergy models have been developed to assess food allergenicity. However, interlaboratory differences in the models used with respect to sensitization and elicitation route, choice of adjuvant, clinical signs, genetic background of the animals, housing conditions, and microbiome composition and metabolic activity in the different vivaria often make it difficult to draw generalized conclusions.^5^ It is important to note that almost all models (except genetic models) require adjuvants to trigger sensitization. Therefore, the choice of the adjuvants together with the exposure route are crucial points to consider. In addition, there are numerous in vivo, ex vivo, and in vitro parameters evaluated for the assessment of food allergy. Figure 2 illustrates the types of in vivo (inside a living organism) or ex vivo (outside an organism) methodology and endpoints used in experimental murine models of food allergy. However, there is a need to establish a list of reliable, validated, and effective endpoint parameters to guide researchers working with animal models of food allergy. In this review, we describe a selective list of the most commonly used experimental applied endpoints in food allergies with a focus on milk, egg, and peanut allergens and critically evaluate their applicability for evaluating sensitization potency. Each endpoint was selected and critically described with strengths and limitation based on consortium experience and occurrence in literature.

## 2 MEASUREMENT OF BODY TEMPERATURE

In murine‐type models of food allergy to milk, eggs, and peanuts, a drop in the core body temperature is often observed after repetitive allergen challenge. This change in body temperature is an indicator of anaphylaxis (Table 1). Temperature is measured before and 30 minutes to 1 hour after allergen challenge, but this parameter can also be monitored over time.^21^, ^22^ Animals sensitized to a given food matrix or protein may display a significant reduction in body temperature (0.5‐10°C)^3^, ^4^ compared with that of naive animals. For an adequate level of sensitivity, 5‐16 animals per group should have their temperatures measured using a rectally inserted thermal probe,^29^ but it is also possible to measure changes over time for individual animals using an electronic ID transponder implanted subcutaneously.^11^, ^12^ To refine, improve, and objectify the currently applied manual monitoring methods, an automatic imaging method has been developed.^14^ It involves a noninvasive measurement of the whole‐body surface temperature paired with assessment of activity (see also Data S1 about activity/behavior via camera). Anaphylaxis imaging has been used in three in vivo allergy mouse models for (a) milk allergy, (b) egg allergy, and (c) peanut allergy in proof‐of‐principle experiments and suggests that imaging technology represents a reliable noninvasive method for objective monitoring of small animals during anaphylaxis over time. This method can be useful for monitoring diseases associated with changes in both body temperature and physical behavior.

**Table 1:** Table of studies measuring body temperature in allergic reactions

Mouse/rat model	Allergen	Number of animals	δT°	Therapeutic or preventive strategy	Conditions of measure	System of measurement	Ref.
C3H/HeOuJ	Whey	N = 6‐12	4°C	Prevention with omega‐3 long chain polyunsaturated fatty acids	1 h after challenge	Implantable electronic ID transponder	11
C3H/HeOuJ	OVA	N = 4‐6	5°C	Prevention with prebiotics: scGOS/lcFOS/pAOS	30 min after challenge	Implantable electronic ID transponder	12
BALB/c	Beta‐lact	N = 10	1.7°C	Prevention with ratios of omega‐6 and omega‐3 fatty acids	Before, 30 and 45 min after challenge	Rectal probe	13
BALB/c	Peanut, egg, milk	N = 3‐5	3°C	Anaphylaxis imaging	Monitoring after challenge	Imaging method for whole‐body surface temperature	14
C3H/HeN	Whey	N = 12‐15	4°C	Microbiota composition and allergy protection	Before and 45 min after challenge	Rectal probe	15
BN	OVA	N = 8	3°C	To develop an effective and rapid model of FA in Brown Norway rats	60, 90 and 120 min after challenge	Rectal probe	16
BALB/c	BLG	N = 5	5°C	Nitration process of allergen	Before and 15 and 30 min after iv	Rectal probe	17
BALB/c	OVA	N = 10	0.5°C	Microbiota composition and allergy protection	Before and 5 and 10 min after iv OVA challenge	Rectal probe	18
BALB/c	OVA	N = 5	1.5°C	Anti‐acid medication for risk of food allergy	Before and 15 min after challenge	Rectal probe	19
BALB/c	OVA	N = 10	None	Heat process on allergen	30 min after challenge	Rectal probe	20
C3H/HeOuJ	Whey	N = 10	2‐10°C	Hydrolyze process on allergen	Measurement of temperature over time: 0, 15, 30, 60, 120 min after challenge	A programmable temperature transponder implanted subcutaneously	21
C3H/HeOuJ	Caseinate	N = 6	5°C	Transglutaminase cross‐linked caseinate process	Measurement of temperature over time: 0, 15, 30, 60, after challenge	A programmable temperature transponder implanted subcutaneously	22
BALB/cTac	OVA	N = 5‐10	5°C	Microbiota signature in allergy	Measurement every 5 min: 5‐60 min	Rectal probe coupled to a Physitemp Thermalert Model TH‐5	23
BALB/c	OVA	N = 10	1°C	To develop models of food allergy and oral tolerance	30 min after challenge	Rectal probe	24
C57BL/6	Peanut	N = 4‐10	2°C	Commensal bacteria for allergy protection	After challenge	Rectal probe	25
C3H/HeJ and BALB/c	Peanut	N = 5	3‐5°C	Skin sensitization study	30 min after challenge	Rectal thermometer (WPI Instruments)	26
C3H/HeJ and BALB/c	OVA or peanut	N = 5	4°C	Epicutaneous immunotherapy	30 min after challenge	Rectal thermometer (WPI Instruments)	27
C3H/HeJ	BLG	N = 10	3°C	Pasteurization process on milk allergen	After challenge	Rectal probe	28

### 2.1 Strengths

- The measurement of core body temperature is a cost‐effective, reliable assessment of the allergic reaction.
- Therapeutic or preventative strategies for the reduction of allergic reactions can be easily evaluated.
- Can be used to evaluate the severity of allergic shock and differences between allergens subjected to physical transformations (ie, native versus processed).

### 2.2 Limitations

- The occurrence of anaphylaxis is dependent on the mouse strain used: Balbc or C3H mice are prone to develop anaphylaxis, whereas C57BL/6 or A/J mice necessitate stringent exposure protocols to achieve sensitization.
- The clinical score may be biased as a consequence of the laboratory environment, stress level, animal strain, and technical experimenter.
- A decrease in temperature is only observed after a food/allergen challenge after a previous sensitization event; this endpoint therefore contains no predictive value for the sensitization potential of a food protein.

### 2.3 Technical recommendations

- Using a rectal probe, mice or rats must be acclimated to the experimental room at least 1 hour before starting the temperature measurements to obtain stable values.
- The rectal temperature must be evaluated 10 minutes to 1.5 hours after the challenge.
- The animal temperature can be registered over time using a programmable temperature transponder implanted subcutaneously.

## 3 EVALUATION OF IMMUNOGLOBULINS IN SERUM

**Figure 3:**
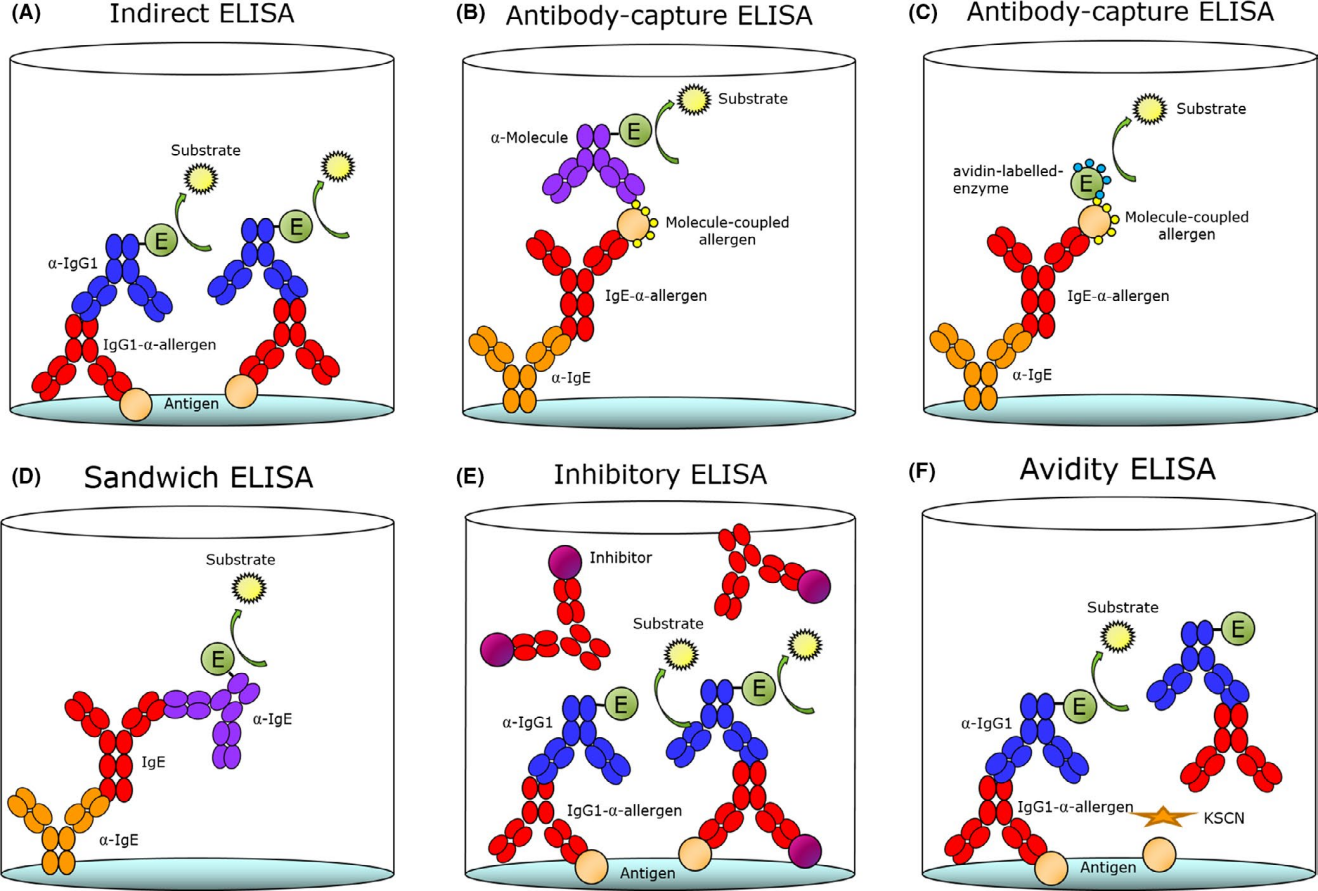
ELISA methods. Antibodies (Abs) can be evaluated by means of different ELISA methods for assessment of their amount, specificity, and avidity. Specific IgG1 Abs are most often analyzed by means of an indirect ELISA (A), while specific IgE is most often analyzed by means of an Ab‐capture ELISA (B, C). Total IgG1 and total IgE are analyzed by a sandwich ELISA (D). Furthermore, the specific Ab responses can also be evaluated for specificity with an inhibitory ELISA (E) or for binding strength with an avidity ELISA (F)

While in vivo measurements are essential to assess the elicitation of an allergic response, they do not provide insight into de novo allergen sensitization. Therefore, blood, tissue, or organs must be collected and further analyzed by ex vivo methods. Serum immunoglobulin (Ig) content is the most common parameter measured when evaluating sensitization to food allergens in animal models, followed by fecal IgA (see Data S1), as antibody responses are considered a direct indicator of allergen sensitization together with mast cell and basophil degranulation. IgE is the most common Ig isotype measured when evaluating the allergenicity of food proteins and is regularly quantified in parallel with IgG1 (Table 2). Total and antigen‐specific Ig levels can be analyzed, where the latter is a measure of how dosing with a given food or protein influences the overall level of IgE or IgG. Serum‐specific IgE and IgG can be quantified by a series of different ex vivo methods, where ELISAs are the most commonly applied, followed by immunoblotting methods and mediator release assays (Figure 3). Whereas specific IgG in general is measured by means of an indirect ELISA,^43^ specific IgE is most often measured by antibody‐capture ELISA.^44^ In fact, IgE is the least abundant Ig isotype in serum (with an approximate amount of only one IgE for every 50 000 IgGs^45^), making it difficult for IgE to compete for binding to proteins coated on ELISA plates. Other methods of measuring specific IgE include enzyme allergosorbent test (EAST) immunoblotting.^34^ When measuring specific IgEs by means of in‐house‐developed antibody‐capture ELISAs, there is a need for coupling the protein of interest to a molecule against which labeled secondary Igs are commercially available, as secondary Igs for direct binding to the proteins of interest can rarely be purchased. Molecules coupled to the protein of interest are most often digoxigenin (DIG)^43^ or biotin,^30^ with the additional advantage that they serve as signal amplifiers (Figure 3). Not only is the total level of specific Igs of interest in evaluating the sensitization response in animal models, the increase in affinity between Igs and the allergen is also important. Studies have shown that the binding strength between specific IgEs and the corresponding allergens is of great importance for the induction of a degranulation response and thereby the severity of the allergic disease.^46^, ^47^ The avidity can be measured by means of simple potassium thiocyanate (KSCN) ELISAs which have shown that no general relationship exists between the level and avidity of specific Igs,^48^, ^49^ though a correlation may be observed during a multiple antigen exposure immune responses. This method, although not very sensitive, is based on the ratio of the areas derived from the curves obtained by plotting the OD and log of the sera dilution in the ELISA experiment with and without thiocyanate treatment. Where measures of specific IgE only allow for evaluation of sensitization, they provide no indication of the biological relevance of the IgEs present in the serum and thereby the clinical relevance of the food allergy model. To provide insights into the biological relevance of secreted IgEs, functional tests should be performed, such as the in vivo temperature drop, a skin prick test (SPT), or evaluation of challenge‐derived symptoms. Further, ex vivo mediator release tests such as the rat basophilic leukemia (RBL) assay and basophil activation test (BAT) enable an evaluation of the biological relevance of the IgE raised in food allergy animal models (see Data S1 for description and opinion about mediator release assays and additional passive cutaneous anaphylaxis (PCA) and active cutaneous anaphylaxis (ACA) models).

**Table 2:** Studies using measurements of Igs from serum

Mouse/rat model	Allergen	Sensitization and challenge	Ig measured in serum	Aim of the study	Ref.
BALB/c	OVA	I.g. + CT followed by i.g. challenge	IgG1, IgG2a: indirect ELISA IgE: Ab‐capture ELISA	To elucidate the class of bioactive polyphenols that exhibit a beneficial anti‐allergic effect and to assess whether the protective effect matches the in vivo bioavailable metabolite concentrations	30
BALB/c	OVA,	I.p. followed by i.g.	IgG1, IgG2a, IgA: indirect ELISA	To investigate how thermal processing influences the ability of ovalbumin (OVA) to induce allergic symptoms and immune responses in a mouse model of food allergy	20
C57BL/6J	OVA	Oral (by feeding) + s.c. + alum followed by i.d. (ear test) challenge	IgG1: indirect ELISA IgE: Ab‐capture ELISA	To investigate the potential of hydrolyzed egg and whole egg to induce tolerance by the oral route (ie, by feeding)	31
BN	OVA	I.p. + alum + tBp followed by i.g. challenge	IgG1, IgG2a, IgG2b, IgA: indirect ELISA IgE: Ab‐capture ELISA	To develop an effective and rapid model of food allergy with only one i.p. injection of the allergen with alum together with toxin from Bordetella pertussis (tBp) to promote IgE production and 2 wk later the i.g. administration of allergens	16
BALB/c	WPC, WPH, or BLG	I.p. with BLG + alum followed by oral (solution in drinking bottle) challenge with WPC and WPH	IgE: Ab‐capture ELISA	To develop an experimental murine model of food allergy to the cow's protein ß‐lactoglobulin (BLG) that mimics the main clinical characteristics of human disease as well as to examine the allergenic and immunological properties of extensively hydrolyzed whey proteins	32
BALB/c	BLG	I.p. + alum or FCA or FIA	IgG1, IgG2a, IgE: indirect ELISA	To study the sensitizing capacity of BLG and the influence of the use of adjuvant.	33
BALB/c	Whole milk	I.g. +/− CT followed by i.g. challenge	IgG1, IgG2a: indirect ELISA IgE: EAST (enzyme allergosorbent test)	To map epitopes of the major soybean allergen Gly m 5 that are corecognized by casein‐specific antibodies and to identify a peptide responsible for the cross‐reactivity	34
C3H HeOuJ	WPC, ALA, BLG	I.g. + CT	IgG1, IgE: indirect ELISA	To test a panel of high and low allergenic proteins	35
C3H HeOuJ PF	WPC or WPH	I.g. + CT followed by i.g. + i.d. challenge	IgG1, IgE: indirect ELISA	To validate a mouse model for cow's milk allergy to assess the potential allergenicity of hydrolyzed cow's milk‐based infant formulas	21
BN	WPH, BLG	I.p. + alum	IgG2a, IgE: indirect ELISA IgG1: inhibitory ELISA	To provide a thorough analysis of the immunogenicity and allergenicity of hydrolyzed cow's milk proteins for use in infant formulas	36
BALB/c	Extract	I.c. + CpG + CT or + non/CpG + CT followed by i.g. + CT challenge	IgG1, IgG2a, IgE, IgA: indirect ELISA	To evaluate the effect of the application of peanut extract (PE) alone or mixed with CT and unmethylated sequences (CpG) as adjuvant on the intact skin	37
BALB/cJ	Roasted extract or Ara h 1	I.n. or e.c. followed by i.g. + CT challenge	IgG1: indirect ELISA IgE: Ab‐capture ELISA	To assess the impact of repeated short‐term epicutaneous (e.c.) applications on intact skin or after repeated intranasal (i.n). administration of food allergens from roasted peanut	38
BALB/c	Ara h 1, Ara h 2, Ara h 3, Ara h 6, and Ara h 6 with no S‐S bridges	I.p. + alum	IgG1, IgE: indirect ELISA (protein G for IgG removal)	To investigate the impact of heat processing of peanut seed on the sensitization to native Ara h 6	39
C3H/HeJ	Extract	I.g. + CT followed by i.p. challenge	IgG1, IgG2a: indirect ELISA IgE, IgA: Ab‐capture ELISA	To reveal the immune responses that are induced against peanuts allergens during sensitization, including the very early responses	40
C3H/HeJ	Whole peanut	I.g. + CT followed by i.g. challenge	IgE: indirect ELISA	To develop a murine model of IgE‐mediated peanut allergy that closely mimics human peanut allergy	41
BN	Ara h 1	I.p.	IgG1, IgG2a: indirect ELISA and inhibitory ELISA for IgG1 IgE: Ab‐capture ELISA Total IgE: sandwich ELISA	To study the sensitizing capacity of four different 7S proteins and to determine whether related proteins would induce similar sensitization when removed from their “normal” matrix	42
BN	Ara h 1	I.p.	IgG1, IgG2a: indirect ELISA IgE: Ab‐capture ELISA	To investigate the ability of digested protein—Ara h 1 to sensitize	43

### 3.1 Strengths

- Specific IgE antibody analysis is the most trustworthy measure of sensitization.
- Measures of specific IgE antibodies are often used to evaluate not only sensitization but also the potential severity of the allergic reaction after a second encounter.
- Measurements of antibodies can be performed without the use of advanced equipment such as a cytometer or robotics.

### 3.2 Limitations

- Assays often need to be developed in‐house, restricting the possibilities for comparison between laboratories.
- IgE only accounts for a fraction of all serum antibodies, requiring more advanced ELISAs for analysis of specific IgE.
- IgE levels do not predict the clinical severity of a food allergy model, and other ex vitro experiments are needed to further address this parameter.
- Measures with optical density (OD) as the unit only allow for one serum dilution.

### 3.3 Technical recommendations

- Antibody‐capture ELISAs should be used for the measurement of specific IgE.
- Other antibody parameters in addition to the amount of total and specific antibodies are relevant and should be measured, such as clonality and avidity.
- Measures of total and specific antibodies should always be expressed as titer values or as concentrations with no upper or lower limit for dilutions.
- Serum depleted of IgG using protein G columns before use in indirect ELISAs needs to be considered.

## 4 PHENOTYPING OF T‐CELL POPULATIONS

Assessment of serum Ig levels provides important information about the sensitization phase but does not allow for quantification of immune cell responses, including cellular infiltration to sites of allergic inflammation. The phenotyping of innate (eg, macrophages, eosinophils, basophils, neutrophils, dendritic cells) and adaptive (B and T cells) responses is indispensable for assessing the mechanisms of allergic sensitization (Table 3). Immune cells are generally isolated from organs, including the mesenteric lymph nodes, spleen, lung, skin, or intestine, and analyzed by flow cytometry. Typically, allergic inflammation is characterized by a predominantly type 2 immune response and secretion of the canonical type 2 cytokines IL‐4, IL‐5, IL‐9, and IL‐13 by innate immune cells (eg, eosinophils, basophils, mast cells (MCs), type 2 innate lymphoid cells, and polarized Th2 cells).^44^, ^45^ Indeed, in mice specifically expressing the ovalbumin T‐cell receptor, sensitization to ovalbumin in their diet induced the expansion of IL‐4‐producing CD4^+^ T cells in mesenteric lymph nodes, the spleen, and Peyer's patches.^60^ Importantly, adoptive transfer of antigen‐specific CD4^+^ T cells derived from mesenteric lymph nodes of OVA‐sensitized mice is sufficient to transfer allergen‐induced diarrhea to naïve recipients. The recipient mice also display an upregulation of the Th2‐related chemokines CCL17 and CCL22 in the small intestine.^61^ In addition to polarized Th2 responses, the proportion of other common T‐cell subtypes, such as Th1 and Th17 that are characterized by the production of IFN‐γ and IL‐17, respectively, can also be elevated in lymphoid organs of allergic mice. In contrast, expansion and/or the regulatory capacity of CD25^+^ Foxp3^+^ T cells associated with tolerance are often compromised in many food allergy models.^62^ Additionally, other T‐cell subtypes can be involved in food allergy pathogenesis. The recently discovered Th9 subset and associated IL‐9 secretion were found to be involved in food allergy and especially in peanut allergies.^63^ IL‐9 is mainly responsible for the production of IL‐4 by Th2 cells to promote mucosal mast cell accumulation and secretion of mucus and chemokines by epithelial cells to sustain allergic inflammation.^64^ To a lesser extent, γδT cells found in the intestinal epithelium and in the lamina propria were also shown to be involved in food allergy. These cells are involved in blocking the induction of tolerance and modulating inflammatory responses.^65^,^66^

**Table 3:** Studies using immune infiltrate as readout of allergic inflammation to egg, milk, and peanut proteins

Mouse/rat Model	Allergen	Allergen sensitization	Allergen challenge	Immune infiltrate	Method	Therapeutic/preventive strategy	Ref.
C57BL/6 or BALB/c	OVA, crude peanut extract	Skin	Intragastric	Mast cells, eosinophils	Flow cytometry for intestinal eosinophils/mast cells, chloroacetate esterase staining for mast cells in jejunum, H&E staining	Anti‐TSLP treatment or basophil depletion limits food allergen sensitization and the development of intestinal food allergy	50
C57BL/6	OVA	Skin	Intragastric	Mast cells, eosinophils	Flow cytometry for intestinal eosinophils/mast cells, chloroacetate esterase staining for mast cells in jejunum, H&E staining	Targeting basophil‐derived IL‐4 reduces food allergen sensitization and limits intestinal food allergy	7
BALB/c	Raw or roasted peanut extracts	Skin	Intragastric	Eosinophils	Flow cytometry for eosinophils in the small intestinal lamina propria; H&E staining jejunum	NA	51
BALB/c	OVA	Skin	Intragastric	Eosinophils	Flow cytometry for peripheral eosinophils; H&E staining jejunum	Basophil depletion attenuates intestinal allergy; CD4 T‐cell depletion limits TSLP‐mediated intestinal food allergy	52
BALB/c	OVA	Systemic	Intragastric	Mast cells, CD4 T cells	H&E staining jejunum, chloroacetate esterase staining of mast cells in the jejunum; flow cytometry of CD4 + T cells in the small intestinal lamina propria	Treatment with mast cell stabilizing cromolyn sodium protects against food allergen sensitization	53
BALB/c	OVA	Skin	Intragastric	Mast cells	Chloroacetate esterase staining of connective tissue mast cells in the jejunum	Targeting of IgE responses prevented intestinal mast cell expansion and anaphylaxis	54
Various	OVA	Intestine	Intragastric	Allergen‐specific Tregs that acquire Th2 mast cells	Flow cytometry of small intestinal Foxp3 + Tregs; chloroacetate esterase staining of connective tissue mast cells in the jejunum	NA	55
BALB/c	OVA; whole peanut extract	Intestine	Intragastric	Mast cells; eosinophils	H&E staining jejunum; pinacyanol erythrosine staining to determine mast cell numbers and granulation status in the jejunum	NA	56
BALB/c	OVA	Skin	Intragastric	Mast cells	Chloroacetate esterase staining of mast cells in the jejunum	ST2 blockade attenuates food‐induced anaphylaxis	57
C3H/HeJ; BALB/c	OVA; ground peanut	Skin; systemic	Intragastric	Lap + Tregs	Flow cytometry for Lap + Tregs in the lamina propria	Clinical protection induced by epicutaneous immunotherapy (EPIT)	58
BALB/c	BLG	Intestine	Intragastric	Lamina propria lymphocytes	ELISPOT IL‐12, IL‐17 producing lymphocytes from the intestinal lamina propria	Blocking TSLP signaling prevents food allergy	59

### 4.1 Strengths

- Precise mechanistic insights into the cellular response in isolated organs and tissues support the sensitizing potential of food proteins when combined with additional readouts.
- Precise determination of the T‐cell profile by using specific markers of the T‐cell population.
- Quantitative evaluation of the infiltrating cell population by flow cytometry.

### 4.2 Limitations

- Analysis of cell populations without the contribution of neighboring cell tissue (loss of microenvironment).
- Isolation of immune cells from tissues relies on enzymatic digestion protocols and may thus alter phenotypical and functional properties of the cells of interest.
- Difficulty with the separation of minor subpopulations.
- Sacrifice of the animal is required for organ and tissue sampling.
- Need for sophisticated equipment such as FACS.
- Type 2 immune response‐associated mucus production in tissues makes cell isolation difficult and can create bias in cell phenotyping and frequencies.

### 4.3 Technical recommendations

- Remove fat and store organs, tissues and cells at 4°C to avoid uncontrolled cell death or degradation of surface markers.
- Perform flow cytometry and culturing the same day as the animal kill.
- Phenotyping of T cells can be achieved by intracellular cytokine/transcription factor staining using flow cytometry.

## 5 CYTOKINE PRODUCTION IN RESPONSE TO ALLERGEN RESTIMULATION

The logical follow‐up to the analysis of infiltration/expansion of innate and adaptive immune cells in the tissues and organs is the evaluation of cytokine secretion. This evaluation comes directly from serum or from lymphatic tissue cells restimulated ex vivo. Food allergen stimulation of only lymphatic tissue cells, or in coculture with dendritic cells, allows for the immunophenotyping of the immune cell populations specific for the exposed food antigen or matrix. To confirm allergen specificity, splenocytes, mesenteric lymph node cells, or lamina propria cells isolated from sensitized and/or challenged mice are restimulated with corresponding allergenic proteins or peptides. After culture for up to 5 days, cytokines associated with the inflammatory response (IL‐4, IL‐5, IL‐13, IL‐17, and IFN‐γ) and the regulatory response (IL‐10 and TGF‐β) are analyzed in the supernatants by ELISA^60^, ^61^, ^62^, ^63^, ^64^ or a multiplex system. The cytokine production indicates whether T cells were primed toward the challenged food proteins and distinguishes Th1 or Th2 cell type responses. The prototypical type 2 cytokines include IL‐4, IL‐5, and IL‐13. While IL‐4 is critical for the polarization of Th2 cells and IgE class‐switching in B cells,^64^ IL‐5 promotes the activation, proliferation, and survival of eosinophils, and IL‐13 induces mucus production from goblet cells. Additional assays may be used including proteomics and gene expression profiling by PCR or microarray technology, that provide mechanistic insights and potential drug targets.

### 5.1 Strengths

- Precise assessment of the allergen specificity by restimulating cells with the same allergen used in the animal model.
- Class determination of the T‐cell response by evaluation of cytokine production in the supernatant of sorted T cells.
- Higher production of cytokines can be obtained after proliferation and restimulation with the antigen than by direct measurement in serum.

### 5.2 Limitations

- Restimulation with allergens can activate nonspecific T cells due to certain cross‐reactivity.
- Difficult to obtain a level above the sensitivity threshold with cells isolated from naïve mice.
- Some mechanistic endpoints are not equally important in animals and humans.

### 5.3 Technical recommendations

- For allergen presentation, presorted T cells need to be co‐cultured with dendritic cells.
- MHC peptide—tetramers can be used to sort specific T cells and have better assessment of allergen specificity.
- Need for positive (polyclonal anti‐CD3/anti‐CD28) and negative control (nonallergen) stimuli to ensure proper T‐cell responsiveness.
- Endotoxin levels within the allergen extract need to be controlled to prevent bias in restimulation responses.
- Ideally, when using gene expression sequencing data, this method should be confirmed with at least one other technology (eg, flow cytometry).
- As cells and mediators associated with immune responses change rapidly, longitudinal assessments of mechanistic endpoints will be more informative than single time point assessments. The timing of the measurements will depend on the research question, for example, sensitization mechanisms vs mechanisms of acute allergic responses following (re)challenge.

## 6 FUTURE ANALYSIS OF FOOD ALLERGY MODELS

To date, the methods to study intestinal pathophysiology are in vitro culture systems with cell lines or explanted mucosa grown in monolayers,^67^, ^68^ intestinal organoid cultures,^69^, ^70^ and “gut‐on‐a‐chip” devices.^71^, ^72^ These technologies have offered many insights into gut physiology, but they lack cellular complexity, architecture, and immune and inflammatory responses that are crucial for a comprehensive understanding of underlying disease mechanisms and pathways. Alternatively, in vivo animal models provide the intact organ in the context of the vascular supply, systemic mediators, and circulating cells. However, in vivo experiments may be hampered by technical difficulties, including interindividual variability and maintenance of constant and reproducible experimental conditions.^5^ To address the limitations of in vitro and in vivo models of gut disease, Yissachar et al^73^ developed a chamber unit for culturing 12‐ to 14‐day‐old mouse colon or small intestine segments under highly controlled conditions. Of particular interest is that the chamber unit has two paired inputs and outputs that allow for controlled introduction of molecules or microbes into the lumen while simultaneously introducing continuous replenishment of medium to support tissue viability. The tissue remains intact, and the overall structure with epithelial cell layers is preserved for at least 24 hours, making this method suitable for studying epithelial transport of food allergens and their effect on epithelial integrity. However, other measurements are currently difficult due to the very short time that such tissue explants can be maintained. Furthermore, the enteric nervous system structure is maintained, and immune cells are detected as they found in healthy intestinal biopsies. It is possible to envisage the use of this type of ex vivo chamber unit in food allergy research by using intestinal fragments from naive, sensitized, and allergic animals to introduce a variety of food proteins. It is thus possible to further elucidate pathways involved in luminal physiology and antigen uptake and presentation and make comparisons between known allergenic and nonallergenic proteins. This approach may lead to novel insights into new proteins and cross‐reactive proteins and to the development of a predictive model for food allergy. Additional studies related to the survival and growth of anaerobic and aerobic microbiota revealed that the ex vivo colonization of cultured tissue with selected microbes may be possible. Indeed, changes in the composition and metabolic activity of gut microbes can influence all aspects of innate and adaptive immune processes within the mucosa (see also Data S1 for stool consistency as a readout in food allergy assessment). Thus, focusing on the effect of diverse microbiota profiles and specific bacteria on immunological responses upon the introduction of allergenic proteins may lead to novel mechanisms, therapeutic targets, or predictive models. However, intra‐ and interlaboratory variability in microbiome composition and metabolic activity after birth as a result of the breeding environment is also a major underlying cause for conflicting results between experiments. This variability must be taken into account beforehand in the experimental design of an animal trial.^5^ It is also noteworthy to consider the possible development of highly controlled chamber units for food allergy research used in combination with in vivo models to provide a new powerful strategy for studying mechanisms in the intestine.

### 6.1 Strengths

- The tissue structure, cellular components, and neural system are highly preserved.
- The model provides the possibility to study immediate responses generated after the introduction of different molecules and microbes.

### 6.2 Limitations

- Only short‐term responses can be evaluated due to changes that can occur in the tissue over time.
- Currently, only intestinal segments from 12‐ to 14‐day‐old mice have been tested.
- Tissue preparation and assembly require specific skills.

## 7 CONCLUSION

The recent broadening of our knowledge of food allergy pathogenesis and development of murine food allergy models has enabled us to model the allergic elicitation reaction as well as the preceding sensitization events and observe relevant symptoms with different food proteins (milk, egg, and peanut). The principal endpoint parameters described in this review are critical parameters that should be evaluated in a correct manner so that they may be powerful in the different rodent models.

Characterizing a food allergy model using temperature, level of Igs, phenotyping of the cell infiltrate, and cytokine production gives an overview of the reaction while providing us insight into the degree of sensitizing capacity of the allergen used. Nevertheless, even though the in vivo measurements and the ex vivo experiments provide us with many answers about the immune response and the sensitization phase, we still do not have a complete overview of the immune mechanisms behind each reaction. There is still a strong need to better define the allergic reaction to predict the clinical outcomes of sensitization to novel food proteins. Although the current available models are suitable for studying the pathophysiology of food allergy, they still cannot predict the magnitude of the allergic potential of a particular allergen. Discovering and highlighting the molecules and cells involved in both sensitization and elicitation are necessary to improve risk assessment models and to facilitate the introduction of novel protein sources into our diet with a low risk of allergic sensitization.

## CONFLICTS OF INTEREST

Dr Blanchard reports other from Nestec, outside the submitted work; Dr O'Mahony reports personal fees from AHL and grants from GSK, outside the submitted work. The other authors declare no conflict of interest.
