# Supplementary material for: Overview of in vivo and ex vivo endpoints in murine food allergy models: Suitable for evaluation of the sensitizing capacity of novel proteins?
Source: Allergy. 2019 Jul 9;75(2):289–301. doi: 10.1111/all.13943 (PMC7065134; doi:10.1111/all.13943)
Supplement: Supplementary file 1 [file ALL-75-289-s001.docx]

**Supplementary section**

**Overview of *in vivo* and *ex vivo* endpoints in murine food allergy models: Suitable for evaluation of the sensitizing capacity of novel proteins?**

Laure Castan^1^, Katrine L. Bøgh^2^, Natalia Z. Maryniak^2^, Michelle M. Epstein^3^, Sahar Kazemi^3^, Liam O’Mahony^4^, Marie Bodinier^1^, Joost Smit^5^, Jolanda van Bilsen^6^, Carine Blanchard^7^, Robert Głogowski^8^, Hana Kozáková^9^, Martin Schwarzer^9^, Mario Noti^10^, Nicole de Wit^11^, Grégory Bouchaud^1‡^, Shanna Bastiaan-Net^11‡^

In this supplementary section, the *in vivo* and *ex vivo* endpoints not elaborated on or discussed in the main text are listed to complete the entire overview of murine model endpoints described in the literature for the allergenic foods milk, eggs and peanuts. Endpoints that are not often used and described in the literature, such as skin-specific responses, weight loss, ear swelling, flexiVent and plethysmography respiratory response measurements, and intestinal permeability responses have not been included in this supplementary section because no critical opinion could be given for these readouts.

**Activity/behavior via camera**

Behavioral changes as a result of a specific food allergen exposure in mice may be evaluated indirectly and typically includes recording of scratching or loss of mobility via camera. Such a noninvasive method to measure physical behavior without disturbing the animals allows for more objective analyses, without the human observer bias reported for the classical score system requiring subjective validation by the investigator **(Table S1)**.

*Strengths*

- *Rapid assessment of allergic responses*
- *Provides more information than just sensitization potential as allergy effector mechanisms become activated*
- *Enables continuous data generation over a prolonged period of time, allowing longitudinal studies within one animal*

*Limitations*

- *Readouts are often subjective*
- *Not all observed responses are allergy-related and IgE-dependent*
- *The experimenter may easily bias manual counting of movements*

*Technical recommendations*

- *This endpoint requires an actual food/allergen challenge to observe sensitization events; this method has no predictive value for allergen sensitization*

**Diarrhea and stool consistency**

Diarrhea is a classic clinical feature of food allergies, including peanut, egg and milk allergies. Oral allergen-induced diarrhea is mostly studied in OVA-challenged allergic mice **(Table S2)**. In this model, systemic sensitization with OVA followed by an oral OVA challenge leads to OVA-specific diarrhea. Diarrhea is mostly assessed by visual monitoring of the mice up to 60 minutes after oral allergen administration and is defined as watery or liquid stool. Generally, there is no diarrhea observed during the sensitization phase, which only becomes apparent in the effector phase after the re-exposure to Ag that triggers the allergic response. The development of diarrhea requires the absorption of Ag, similar to that required to trigger an anaphylactic shock. Mast cells seem to play an important role in allergen-induced diarrhea, especially by synergistic signaling via serotonin and platelet-activating factor (PAF) (74). This mast cell activation by oral Ag challenge linked to diarrhea seems to be a local (intestinal) effect, as diarrhea can occur without systemic shock. Furthermore, there is increasing evidence that gut microbiota can influence allergenic diarrhea and that this can be potentially modulated by probiotics (75,76).

*Strengths*

- *Allows for an easy and rapid assessment of allergic elicitation reactions*
- *Apparent at low dosages of allergens (lower than needed to trigger shock)*

*Limitations*

- *Scoring is subjective to observer bias*
- *Assessment of watery stool is a readout of diarrhea that is not quantitative*

*Technical recommendations*

- *No predictive value for allergen sensitization as diarrhea only becomes apparent in the effector phase*

**Anaphylaxis models (PCA/ACA)**

Two models to measure anaphylactic responses exist, namely, the cutaneous and systemic anaphylaxis models **(Table S3)**. The latter was discussed in the main manuscript. In cutaneous anaphylaxis models, mice are sensitized by several injections with an allergen. In the passive cutaneous anaphylaxis (PCA) model, serum from these sensitized animals is injected into the dermis of naïve recipient mice, after which they are intravenously (i.v.) challenged with the allergen mixed with a dye. Because of the increased capillary permeability due to the local mast cell-IgE cross-linking and histamine release, extravasation of the dye is observed with the subsequent formation of a blue spot, which can be quantified. In the active cutaneous anaphylaxis (ACA) model, sensitized mice are i.d. challenged with allergen combined with an i.v. injection with dye to quantify localized allergic responses by using dye extravasation. Historically, a variety of experimental methods have been used, including injection of antigen prior to injection of the dye, injection of dye prior to injection of the antigen, and simultaneous injection of the dye and antigen. I.v. dye administration as a means for measuring allergic responses is a versatile assay because it can be used for measuring active, passive, and reverse passive local reactions. Numerous dyes have been utilized to assess allergic responses, including Trypan Blue, Pontamine Sky Blue, Evan’s Blue, Geigy Blue 536, and India Ink. A solution of 0.5% Evan’s Blue is currently the standard dye used for measuring allergic responses in the skin. Quantification of dye extravasation was originally obtained by measuring wheal size. Additionally, colorimetric analysis of recovered dye from the ears or counts of degranulated mast cells can be quantified by excising skin tissue from the site of the reaction and staining with toluidine blue.

*Strengths*

- *Blue dye extravasation in ACA and PCA enables both visualization and relative quantification of a localized allergic reaction*
- *Animals are usually euthanized shortly after challenge, limiting any discomfort potentially caused by the allergen challenge (+/- dye administration)*

*Limitations*

- *I.v. and i.d. injections are technically challenging*
- *Evan’s Blue dye is toxic*
- *The results are subjective to observer bias*
- *IgG1, IgG2a and IgG2b (but not IgG3) can induce PCA in mice, whereas in humans, there is no definitive evidence to date*
- *The anaphylaxis models are not suitable to predict sensitization because an actual food/allergen challenge is required*

*Technical recommendations*

- *Reliable results are dependent on consistent and successful ear and tail vein injections; if the entire challenge dose is not administered into the ear, if ear injection results in obvious puncture of a blood vessel, or if the Evan’s Blue i.v. injection is not complete, the animal should be excluded from analysis*
- *PBS injection in ACA/PCA should routinely be used as an internal control*
- *Injection to the back skin of mice may be less technically challenging, and, in addition, it allows for the testing of multiple antigens simultaneously*

**Basophil/mast cell activation test (BAT)**

Mast cells (MCs) are innate immune cells derived from CD34^+^ progenitor stem cells in bone marrow (77,78). MCs enter circulation as immature cells and undergo maturation within peripheral tissues, such as the skin and gastrointestinal tract. Maturation and homing are strongly affected by the local tissue microenvironment, the presence of stem cell factor and lipoteichoic acid and critical MC transcription factors that regulate phenotype and cell fate (79,80). Antigen-specific IgE cross-linking on MCs leads to the release of proteases and various inflammation mediators, such as histamine, prostaglandins and serotonin. Serum mast cell protease MCPT-1 is commonly used as a systemic readout for MC activation in mouse food allergy models, although its levels may vary depending on the genetic background. Factors influencing MC tissue homing and methods used for MC analysis are described in **Table S4.** Basophils are granulocytes developed from CD34+ multipotent hematopoietic stem cells that, contrary to MCs, undergo maturation in the bone marrow (81). Basophils, similar to MCs, rapidly secrete allergic inflammatory mediators upon allergen-IgE cross-linking of Fc fragments on their cell surface. The basophil activation assay (BAT), currently used primarily in humans instead of murine allergy models, has been suggested as a new diagnostic test for food allergy with the aim of replacing the oral food challenge, currently the gold standard for food allergy diagnosis in humans (82). The BAT is an *in vitro* flow cytometry-based functional test that determines the degree of basophil activation after exposure to specific allergen stimuli by changes in cell surface membrane markers such as CD63, IgE/CD203c, CCR3/CD3, CD123/HLA-DR (83), which can be rapidly assessed to measure human basophil activation. In contrast to IgE-mediated anaphylaxis in humans, both IgG and IgE can play a role in murine anaphylaxis. Murine specific basophil activation markers include CD200R1 (84) and CD41(85) for IgE-mediated activation, while CD200R3 can be used as an IgG1-specific activation marker (86). Its sensitivity and specificity differs for different allergens, and the standardization of results between laboratories is still challenging (82,84,87).

*Strengths*

- *MCPT-1 is generally used as a systemic readout of MC activation in mouse food allergy models*
- *The BAT has good diagnostic accuracy in food allergy diagnosis without the need to perform stressful and sometimes fatal oral food challenges*
- *Detection of β-hexosaminidase in MCs can be performed colorimetrically, which is more favorable in terms of costs compared to those of an ELISA*

*Limitations*

- *Mature MCs are limited to tissues and hardly circulate in peripheral blood, which makes it difficult to obtain sufficient quantities for in vitro tests*
- *MCPT-1 induction may vary with the mouse genetic background; combination with other readouts is envisaged to describe anaphylactic status*
- *BAT standardization between laboratories is challenging*

*Technical recommendations*

- *The BAT assay needs to be performed within 4 hours of drawing blood*

**Mucus/fecal IgA**

Early studies proposed fecal IgA as a noninvasive biomarker of food tolerance. Often measured as total IgA, the specificity of the effect toward allergen uptake is debatable. A decrease in specific secretory IgA has been observed in most food allergy models **(Table S5),** but specific secretory IgA is not required for oral tolerance acquisition after food exposure in the absence of sensitization (88). Interestingly, this finding may be because the development of allergen-specific anti-OVA IgA in intestinal lavage seems to require oral exposure and (oral) sensitization since intraperitoneal sensitization alone does not seem sufficient to mount such an intestinal-specific IgA response (33,44). While the level of intestinal lavage IgA varies in function of the allergen used for sensitization, IgG1 and IgE alone or combined seemed to have more discriminating potency for different allergens or processing of allergens (milk proteins). Notably, the presence of IgA in feces is highly linked to the gut microbial composition (44). In a peanut allergy model, mucosal IgA responses induced by commensal bacteria were essential in preventing intestinal inflammation; fecal IgA might thus be a potential contributor of gut microbiota-mediated immune exclusion, possibly reducing systemic allergen uptake (51).

*Strengths*

- *IgG1 and IgE alone or combined seem to have more discriminating potency for different allergens or processing of allergens*
- *Fecal IgA as a potential contributor to gut microbiota-mediated immune exclusion*

*Limitations*

- *The presence of IgA in feces is highly linked to the gut microbial composition*

*Technical recommendations*

- *Fecal/intestinal total or specific IgA could be used as additional markers of interest for allergen sensitization capability but are not sufficient today as standalone markers*

**Table S1:** Literature covering activity/behavior via camera.

| **Mouse/rat model** | **Allergen** | **Number of animals** | **Therapeutic or preventive strategy** | **System of measurement** | **Conditions of measure** | **Ref** |
| --- | --- | --- | --- | --- | --- | --- |
| C3H/HeOuJ | Whey | N=20 | Effects of an immune response on behavior and neuronal activation | Video tracking software | 24 and 48 h after last sensitization and 24 h after challenge | (89) |
| BN rats | OVA | N=24 | Development of an effective food allergy model | Activity meter recording movements for a 21-min period | 24 h before induction and immediately after challenge | (44) |
| C3H/HeN | BLG | N=30 | Impact of gut microbiota on allergic manifestations | Scratching and mobility loss scoring by protocol-blinded investigators | 15-45 min. after challenge | (43) |
| BALB/c | Cow's milk | N=18 | Cross-reactivity *in vivo* study using a food allergy model | Responses assessed by two independent investigators | 30 min. after challenge | (90) |
| BALB/c | OVA | N=20 | Development of food allergy and oral tolerance models | Reactions monitoring by two independent individuals | between 30 to 60 min. postchallenge | (88) |
| BALB/c | OVA, peanut | N=6-8 | Testing the immunologic mechanisms of intestinal food allergy predisposition by epicutaneous sensitization | Clinical symptoms scoring investigated by treatment-blinded investigators (0-6) | Individual observations for 30 min. starting 20 min. after challenge | (64) |
| BALB/c | Peanut, cow milk, potato | N=32 | Food protein extracts allergenicity evaluation | Monitoring using symptoms pre-established scale | 10 min prior and in 10 min. intervals (up to 50) after the challenge | (91) |
| BALB/c | Casein, peanut, OVA | N=32 | Automated noninvasive physical activity imaging | 20 min. of video camera imaging | Immediately after challenge | (16) |
| C3H/HeJ | Peanut | N=5 | Immunotherapy with allergen nanoparticles | Visually observed symptoms scoring (0-5) | 30 to 40 min. after challenge | (92) |
| BALB/c | Lactoferrin | N=60 | Dose-dependent allergenic properties measurement | Visually observed symptoms scoring (0-5) | 30 min. after injection | (93) |

**Table S2:** Studies using diarrhea as a readout for allergic reactions.

| **Mouse/rat model** | **Allergen** | **Number of animals** | **Conditions of measure** | **System of measurement** | **Ref** |
| --- | --- | --- | --- | --- | --- |
| BALB/c | OVA | N=5-6 | 1 h after challenge | Diarrhea was assessed by visually monitoring mice. Mice with profuse liquid stool were recorded as diarrhea-positive animals. | (94) |
| BALB/c | OVA | N=15-16 | 1 h after challenge | Diarrhea was assessed by visually monitoring mice. Diarrhea was defined as the development of watery green stools. | (74) |
| BALB/c | OVA | N=5 | 1 h after challenge | Diarrhea was assessed by visually monitoring mice. Diarrhea was defined as the development of watery green stools. | (95) |
| BALB/c | OVA | N=12 | 1 h after challenge | Diarrhea was assessed by visually monitoring mice. Mice with profuse liquid stool were recorded as diarrhea-positive animals. | (75) |
| BALB/c | OVA | N=27-34 | Not specified | Not specified | (76) |

**Table S3:** Studies implementing anaphylactic models as a readout for allergy sensitization.

| **Mouse/rat model** | **Allergen** | **Number of animals** | **Sensitization** | **Challenge** | **Conditions of measure** | **System of measurement** | **Ref** |
| --- | --- | --- | --- | --- | --- | --- | --- |
| BALB/c | Peanut agglutinin; OVA | N=5 | I.d. pooled allergic serum samples in ears of naïve recipient mice | After 48 hours: i.v. allergen + Evan’s Blue dye | 30 minutes after challenge | Dye extravasation | (96) |
| BALB/c | Peanut agglutinin; OVA | N=5 | I.d. pooled allergic serum samples in ears of naïve recipient mice | After 48 hours: i.v. allergen + Evan’s Blue dye | 30 minutes after challenge | Dye extravasation | (97) |
| BALB/c | BLG, Ara h1, Ara h2 | N=5 | I.d. allergic serum samples in ears of naïve recipient mice | After 48 hours: i.v. allergen + Evan’s Blue dye | 30 minutes after challenge | Dye extravasation | (98) |
| BALB/c | OVA | N=15 | I.p. OVA with alum | S.c. injection with OVA, followed by i.v. injection with Evan’s Blue dye | 30 minutes after challenge | Dye extravasation | (99) |
| ICR | BSA | N=5 | S.c. daily injection with D-galactose for 6 weeks | I.d. injection with BSA in shaved back & i.v. Evan’s Blue dye | 20 minutes after challenge | Dye extravasation | (100) |
| BALB/c | OVA | N=5-6 | 7x i.p. OVA with alum | I.v. injection Evan’s Blue, followed by i.d. injection of OVA in ears | 30 minutes after challenge | Dye extravasation | (101) |
| BALB/c | OVA | N=9-10 | I.p. OVA with alum (IgE) or i.p. OVA with CFA (IgG1) | I.d. ear injection of OVA + i.v. injection of Evan’s Blue | 30 minutes after challenge | Dye extravasation | (102) |
| 129/Sv | TNP-OVA | N=5-7 | I.v. IgE anti-TNP | After 24 hours: i.v. injection of TNP_4_-OVA | 0 – 60 minutes after challenge | Clinical symptoms of anaphylaxis | (103) |
| C57BL/6J | OVA | N=7-15 | 6x OVA i.p. | I.p. injection of OVA | 0 – 120 minutes after challenge | Clinical symptoms of anaphylaxis | (104) |
| ICR | BSA | N=5 | S.c. daily injection with D-galactose for 6 weeks | I.v. injection with BSA | 0 – 60 minutes after challenge | Clinical symptoms of anaphylaxis | (100) |

**Table S4:** Studies and methods used for analyzing mast cell (MC) activation and recruitment.

| **Mouse/rat model** | **Allergen** | **Number of animals** | **Food allergy induction and/or mast cell (MC) detection** | **MC maturation and activation measurement** | **Key messages** | **Ref** |
| --- | --- | --- | --- | --- | --- | --- |
| Germ-free and conventional C57BL/6J,  Kit^W-sh^ | - | Not specified | Histological staining: toluidine blue,  flow cytometry of skin tissue cells stained with anti-CD117, -FcεRI. | *In vivo* MC activation of GF and CV mice using degranulation-provoking compound 48/80.  qPCR on laser-dissected MCs | GF mice have immature MCs in the dermis; skin microbiome drives SCF production in keratinocytes, which triggers the homing and maturation of dermal MCs | (105) |
| BALB/c | OVA | N=6 | Epicutaneous sensitization with OVA followed by i.g. doses of OVA  Immunohistochemical: MCPT-1+ cells | Mast Cell Protease-1 (MCPT-1) in sera by ELISA | Adjuvant-free model of OVA food allergy was established | (106) |
| BALB/c | OVA | N=10 | I.p. sensitization with OVA+alum followed by i.g. doses of OVA | MCPT-1 in sera by ELISA | Thermal processing of OVA decreased its allergenicity | (107) |
| BN rats | OVA | N=20 | I.g. doses of OVA for 6 weeks (adjuvant-free)  Histological staining: toluidine blue,  electron microscopy | qPCR, Western blot, Ca2+ imaging, immunofluorescence of store-operated calcium channels on MCs obtained by peritoneal lavage | OVA-induced food allergy upregulated Ca^2+^ entry through SOCs, thereby leading to subsequent mast cell activation and degranulation | (48) |
| Germ-free*,* conventional and gnotobiotic BALB/c | OVA | N=11-14 | I.p. sensitization with OVA+alum followed by i.g. doses of OVA  Histological staining: pararosaniline,  Flow cytometry of skin tissue cells stained with anti-CD117, -FcεRI. | *In vivo* MC activation of GF and CV mice using degranulation-provoking compound 48/80.  MCPT-1 in sera and jejunum homogenates by ELISA | Germ-free mice don’t develop symptoms of food allergy. Intestinal microbiota is necessary for MC maturation and homing to the gut. | (108) |

**Table S5:** Studies using mucus/fecal IgA measures as a readout for allergy sensitization

| **Mouse/rat model** | **Allergen** | **Number of animals** | **Exposure route** | **Conditions of measure** | **System of measurement** | **Observed effects** | **Ref** |
| --- | --- | --- | --- | --- | --- | --- | --- |
| BN | OVA | N=8 | Intraperitoneal injection vs injection plus oral gavage daily for 3 weeks (5 weeks total) | Serum sIgA at day 0, 14, 21, 28, 35 and 42; gut washes from sacrificed rats 2 days after anaphylactic response; IgA mRNA from small intestine 2 days after anaphylactic response | Indirect ELISA & Taqman-PCR | sIgA levels decreased | (44) |
| BN | OVA | N=10 | Oral gavage daily for 6 weeks | Gut washes and small intestinal tissue biopsies from sacrificed rats 1.5 h after challenge | ELISA | No effects; sIgA increased by probiotics | (109) |
| C3H/HeJ | ALA BLG, casein | Not indicated | Oral gavage on days 0, 7, 14,  21, 28 and 35 | Intestinal lavage IgA content was measured after anaphylactic response challenge on day 42 | ELISA | sIgA levels were decreased | (33) |
| C57BL/6, C57BL/6Foxp3^gfp^ | Peanut | N=4-8 | Intragastric sensitization in combination with antibiotics and/or Clostridia | Fecal IgA in peanut-sensitized mice (pathogen-free, *Clostridium*, *B. uniformis* or conventional treated mice) was measured 24 h after challenge | ELISA | IgA levels were reduced | (51) |

**References**

[74] Brandt EB, Strait RT, Hershko D, Wang Q, Muntel EE, Scribner TA et al. Mast cells are required for experimental oral allergen–induced diarrhea. J Clin Invest 2003;112:1666–1677.

[75] Wang J-H, Fan S-W, Zhu W-Y. Development of Gut Microbiota in a Mouse Model of Ovalbumin-induced Allergic Diarrhea under Sub-barrier System. Asian-Australasian J Anim Sci 2013;26:545–551.

[76] Yang B, Xiao L, Liu S, Liu X, Luo Y, Ji Q et al. Exploration of the effect of probiotics supplementation on intestinal microbiota of food allergic mice. Am J Transl Res 2017;9:376–385.

[77] Kraneveld AD, Sagar S, Garssen J, Folkerts G. The two faces of mast cells in food allergy and allergic asthma: The possible concept of Yin Yang. Biochim Biophys Acta - Mol Basis Dis 2012;1822:93–99.

[78] Dahlin JS, Hallgren J. Mast cell progenitors: Origin, development and migration to tissues. Mol Immunol 2015;63:9–17.

[79] Tshori S, Nechushtan H. Mast cell transcription factors—Regulators of cell fate and phenotype. Biochim Biophys Acta - Mol Basis Dis 2012;1822:42–48.

[80] Kunii J, Takahashi K, Kasakura K, Tsuda M, Nakano K, Hosono A et al. Commensal bacteria promote migration of mast cells into the intestine. Immunobiology 2011;216:692–697.

[81] Yang B, Yang C, Wang P, Li J, Huang H, Ji Q et al. Food Allergen-Induced Mast Cell Degranulation is Dependent on PI3K-Mediated Reactive Oxygen Species Production and Upregulation of Store-Operated Calcium Channel Subunits. Scand J Immunol 2013;78:35–43.

[82] Santos AF, Lack G. Basophil activation test: food challenge in a test tube or specialist research tool? Clin Transl Allergy 2016;6:10.

[83] McGowan EC, Saini S. Update on the Performance and Application of Basophil Activation Tests. Curr Allergy Asthma Rep 2013;13:101–109.

[84] Torrero MN, Larson D, Hübner MP, Mitre E. CD200R surface expression as a marker of murine basophil activation. Clin Exp Allergy 2009;39:361–369.

[85] Bakocevic N, Claser C, Yoshikawa S, Jones LA, Chew S, Goh CC et al. CD41 is a reliable identification and activation marker for murine basophils in the steady state and during helminth and malarial infections. Eur J Immunol 2014;44:1823–1834.

[86] Iwamoto H, Matsubara T, Nakazato Y, Namba K, Takeda Y. Decreased expression of CD200R3 on mouse basophils as a novel marker for IgG1-mediated anaphylaxis. Immunity, Inflamm Dis 2015;3:280–288.

[87] Bridts CH, Sabato V, Mertens C, Hagendorens MM, De Clerck LS, Ebo DG. Flow Cytometric Allergy Diagnosis: Basophil Activation Techniques. In: Methods in molecular biology (Clifton, N.J.). 2014:1192: 147–159.

[88] Perrier C, Thierry A-C, Mercenier A, Corthésy B. Allergen-specific antibody and cytokine responses, mast cell reactivity and intestinal permeability upon oral challenge of sensitized and tolerized mice. Clin Exp Allergy 2009;40:153–162.

[89] de Theije CGM, Wu J, Koelink PJ, Korte-Bouws GAH, Borre Y, Kas MJH et al. Autistic-like behavioural and neurochemical changes in a mouse model of food allergy. Behav Brain Res 2014;261:265–274.

[90] Smaldini P, Curciarello R, Candreva A, Rey MA, Fossati CA, Petruccelli S et al. In vivo Evidence of Cross-Reactivity between Cow’s Milk and Soybean Proteins in a Mouse Model of Food Allergy. Int Arch Allergy Immunol 2012;158:335–346.

[91] Zhou C, Ludmila T, Sun N, Wang C, Pu Q, Huang K et al. BALB/c mice can be used to evaluate allergenicity of different food protein extracts. Food Agric Immunol 2016;27:589–603.

[92] Srivastava KD, Siefert A, Fahmy TM, Caplan MJ, Li X-M, Sampson HA. Investigation of peanut oral immunotherapy with CpG/peanut nanoparticles in a murine model of peanut allergy. J Allergy Clin Immunol 2016;138:536–543.e4.

[93] Negaoui H, El Mecherfi KE, Tadjer SA, Grar H, Kheroua O, Saidi D. Bovine lactoferrin allergenicity as studied in murine model of allergy. Food Agric Immunol 2016;27:711–723.

[94] Kucuk ZY, Strait R, Khodoun M V., Mahler A, Hogan S, Finkelman FD. Induction and suppression of allergic diarrhea and systemic anaphylaxis in a murine model of food allergy. J Allergy Clin Immunol 2012;129:1343–1348.

[95] Kinney SRM, Carlson L, Ser-Dolansky J, Thompson C, Shah S, Gambrah A et al. Curcumin Ingestion Inhibits Mastocytosis and Suppresses Intestinal Anaphylaxis in a Murine Model of Food Allergy. PLoS One 2015;10:e0132467.

[96] Herouet-Guicheney C, Aldemir H, Bars R, de Barbeyrac D, Kennel P, Rouquié D et al. Inter-laboratory comparisons of assessment of the allergenic potential of proteins in mice. J Appl Toxicol 2009;29:141–148.

[97] Dearman RJ, Skinner RA, Herouet C, Labay K, Debruyne E, Kimber I. Induction of IgE antibody responses by protein allergens: inter-laboratory comparisons. Food Chem Toxicol 2003;41:1509–1516.

[98] Dearman RJ, Kimber I. Assessment of Protein Allergenicity Following Systemic Exposure in Mice: Relationship with Immunogenicity. Toxicology 2009;262:1.

[99] Arakawa T, Deguchi T, Sakazaki F, Ogino H, Okuno T, Ueno H. Supplementary seleno-L-methionine suppresses active cutaneous anaphylaxis reaction. Biol Pharm Bull 2013;36:1969–1974.

[100] Park J-H, Choi T-S. Splenocyte proliferation and anaphylaxis induced by BSA challenge in a D-galactose-induced aging mouse model. Cent Eur J Immunol 2016;3:324–327.

[101] Ogino H, Sakazaki F, Okuno T, Arakawa T, Ueno H. Oxidized dietary oils enhance immediate- and/or delayed-type allergic reactions in BALB/c mice. Allergol Int 2015;64:66–72.

[102] Inagaki N, Miura T, Nagai H, Koda A. Active cutaneous anaphylaxis (ACA) in the mouse ear. Jpn J Pharmacol 1992;59:201–208.

[103] Makabe-Kobayashi Y, Hori Y, Adachi T, Ishigaki-Suzuki S, Kikuchi Y, Kagaya Y et al. The control effect of histamine on body temperature and respiratory function in IgE-dependent systemic anaphylaxis. J Allergy Clin Immunol 2002;110:298–303.

[104] Balbino B, Sibilano R, Starkl P, Marichal T, Gaudenzio N, Karasuyama H et al. Pathways of immediate hypothermia and leukocyte infiltration in an adjuvant-free mouse model of anaphylaxis. J Allergy Clin Immunol 2017;139:584–596.e10.

[105] Smit JJ, Willemsen K, Hassing I, Fiechter D, Storm G, van Bloois L et al. Contribution of Classic and Alternative Effector Pathways in Peanut-Induced Anaphylactic Responses. PLoS One 2011;6:e28917.

[106] Wang Z, Mascarenhas N, Eckmann L, Miyamoto Y, Sun X, Kawakami T et al. Skin microbiome promotes mast cell maturation by triggering stem cell factor production in keratinocytes. J Allergy Clin Immunol 2017;139:1205–1216.e6.

[107] Vaali K, Puumalainen TJ, Lehto M, Wolff H, Rita H, Alenius H et al. Murine model of food allergy after epicutaneous sensitization: Role of mucosal mast cell protease-1. Scand J Gastroenterol 2006;41:1405–1413.

[108] Schwarzer M, Hermanova P, Srutkova D, et al. Germ-free mice exhibit mast cells with impaired functionality and gut homing and do not develop food allergy. Front. Immunol. 2019;10:205. doi: 10.3389/fimmu.2019.00205

[109] Huang J, Zhong Y, Cai W, Zhang H, Tang W, Chen B. The effects of probiotics supplementation timing on an ovalbumin-sensitized rat model. FEMS Immunol Med Microbiol 2010;60:132–141.
